# Exploring the Links Between Body Dysmorphic Concerns and Acceptance of Cosmetic Surgery: The Role of Internal and External Shame as Mediators

**DOI:** 10.1111/jocd.71083

**Published:** 2026-08-31

**Authors:** Gabrielle Victoria Topalian, Georges‐Alain Al Tekle, Katia Iskandar, Pascal Lahoud, Feten Fekih‐Romdhane, Souheil Hallit, Sahar Obeid

**Affiliations:** ^1^ School of Medicine and Medical Sciences Holy Spirit University of Kaslik Jounieh Lebanon; ^2^ School of Pharmacy Lebanese International University Beirut Lebanon; ^3^ The Tunisian Center of Early Intervention in Psychosis, Department of Psychiatry “Ibn Omrane” Razi Hospital Manouba Tunisia; ^4^ Faculty of Medicine of Tunis University of Tunis El Manar Tunis Tunisia; ^5^ Applied Science Research Center Applied Science Private University Amman Jordan; ^6^ Department of Psychology and Education, School of Arts and Sciences Lebanese American University Jbeil Lebanon

**Keywords:** acceptance of cosmetic surgery, body dysmorphic concerns, Lebanon, shame

## Abstract

**Introduction:**

Cosmetic surgery has become increasingly normalized in many societies, including Lebanon, where appearance enhancement is often associated with identity, social acceptance, and social mobility. Body dysmorphic concerns (BDC) and shame have both been associated with attitudes toward cosmetic surgery; however, the emotional processes underlying these associations remain insufficiently explored. The purpose of this study is to assess the mediating role of shame in the relationship between BDC and acceptance of cosmetic surgery in Lebanese adults.

**Methods:**

In this cross‐sectional study, 399 Lebanese participants aged 18 years and older were enrolled. Measures used were the Acceptance of Cosmetic Surgery Scale (ACSS), the Dysmorphic Concern Questionnaire (DCQ), a screening measure of subclinical dysmorphic concerns, and the External and Internal Shame Scale (EISS).

**Results:**

Indirect associations statistically consistent with partial mediation models involving total, internal, and external shame were observed between BDC and acceptance of cosmetic surgery. Higher BDC was significantly associated with higher shame and directly associated with higher acceptance of cosmetic surgery. Higher shame was significantly associated with lower acceptance of cosmetic surgery.

**Conclusion:**

These results indicate that the role of shame in crafting attitudes toward cosmetic surgery is rather complicated as an emotional variable. BDC and shame can be screened for during preoperative consultations by clinicians to determine which patients are at risk of having psychological concerns. Applying mental health care to the perspective of aesthetic medicine can increase patient satisfaction and result in an ethical, patient‐centered approach.

## 1 Introduction

Over recent decades, cosmetic surgery has transitioned from being primarily a medical specialty procedure to becoming increasingly normalized within mainstream culture globally [1]. The increased acceptance of cosmetic surgery across the world can be attributed to an overall increase in aesthetic elective treatments, as a result of advancements in technology; however, this is also due to changes in how societies view beauty, identity, attractiveness, and self‐esteem [2]. Studies that specifically address cosmetic surgery in the Middle East have reported prevalence rates of 10% to 13% [3]. These studies have identified various sociodemographic factors that are correlated with undergoing cosmetic surgery including age, education level, and employment status [3, 4]. Individuals who have undergone cosmetic surgery have been found to report a significantly higher rate of mental health symptoms (65.1%) when compared to those who have not (29.8%) [5]; and therefore, there appears to be a significant association between aesthetic practices and psychological wellbeing [6]. Cosmetic surgery is performed for many physical and psychological reasons. Dissatisfaction associated with body image has emerged as a significant contributor to both interest in and acceptance of aesthetic treatments [7]. Body dysmorphic concerns (BDC), which are characterized by a persistent and distressful preoccupation with perceived imperfections in one's appearance, have also been shown to be related to interest in cosmetic surgery [8].

### 1.1 The Relationship Between BDC and Acceptance of Cosmetic Surgery

In the overall body of clinical literature, body dysmorphic disorder (BDD) is defined as a serious mental health disorder that is categorized as one of the obsessive‐compulsive and related disorders [9], characterized by a preoccupation with perceived physical imperfections or deformities [10]. Conversely, BDC represents subclinical levels of concern and distress regarding appearance and are typically measured utilizing screening tools such as the Dysmorphic Concern Questionnaire [11]. While BDC varies among individuals within the general population, higher levels of BDC have been associated with increased body image dissatisfaction and an increased desire for aesthetic alteration [11, 12]. Individuals with high levels of dysmorphic concerns may engage in repetitive appearance‐related behaviors like mirror checking or social comparison, even without having a clinical diagnosis [13]. Research has shown that individuals meeting diagnostic criteria for BDD frequently seek cosmetic procedures, although psychological benefit following surgery often remains limited, suggesting that psychological assessment may be important in aesthetic contexts [14, 15]. While dysmorphic concerns are highly correlated with dissatisfaction with appearance, they do not uniformly predict cosmetic surgery attitudes or behaviors, suggesting that broader socio‐cognitive and emotional processes may shape aesthetic decision‐making [16]. An example of such a mechanism is shame, a self‐conscious feeling associated with negative self‐assessment and fear of rejection from others [17].

### 1.2 Shame as a Mediator

Shame encompasses two related but separate elements: internal shame and external shame [18]. Internal shame describes the critical self‐assessment and perceptions of one's own defects, while external shame reflects an individual's beliefs about the negative judgments or diminished value they believe others have of them [19]. In other terms, internal shame affects self‐directed dissatisfaction, while external shame reflects an individual's sensitivity to others' evaluations [20]. Shame has been consistently linked to the internalization of appearance ideals and increased vulnerability to appearance‐related distress, given that physical appearance is often emphasized as a means to social status and value [21]. In contrast to guilt, which focuses on specific behaviors, shame is self‐directed and typically results in a person avoiding or concealing their flaws and utilizing different coping strategies to minimize the individual's sense of inadequacy [22]; therefore, shame may be associated with greater psychological distress among individuals with BDC and may shape attitudes toward cosmetic surgery in complex ways [23].

In terms of body image models, individuals' concerns regarding their bodies may serve as emotional motivators for their appearance dissatisfaction and stimulate behaviors that are intended to alter how they appear [24]. It is important to understand the influences of internal and external types of shame on cosmetic surgery attitudes in a manner consistent with their unique psychological pathways. One framework that helps explain these processes is Objectification Theory [25], which suggests that repeated exposure to societal appearance standards may promote body surveillance, self‐objectification, and body shame [26]. Research has shown that these processes are associated with appearance‐related distress and body dissatisfaction [27, 28].

Broader sociocultural models have also emphasized the role of appearance‐based pressures in shaping BDC and attitudes toward cosmetic surgery. The Tripartite Influence Model proposes that media, peers, and family contribute to body dissatisfaction through the internalization of appearance ideals and appearance comparison processes [29, 30]. Research has further extended this framework to digital environments, where social media exposure, engagement with appearance‐focused content, and self‐presentation behaviors have been associated with increased body dissatisfaction, body surveillance, and acceptance of cosmetic surgery [31, 32]. These mechanisms can result in increased scrutiny of a person's appearance and susceptibility to emotions related to perceived physical inadequacy. Investigations of appearance‐related activities on Instagram have revealed that idealized and manipulated online images may increase appearance comparison and perceived social evaluation, both of which relate to shame and dysmorphic concerns [33, 34]. The findings imply that shame is probably not a standalone emotion but a socio‐cognitive process that internalizes appearance standards, social comparison, and perceived interpersonal judgment. This interpretation is in line with recent findings indicating that appearance‐focused self‐presentation behaviors may lead to the consideration of cosmetic surgery via body surveillance and body shame processes, reinforcing the larger sociocultural context for the operation of shame‐related mechanisms [35].

Within this broader sociocultural framework, shame may represent an emotional response emerging from internalized appearance standards, social comparison, and perceived interpersonal evaluation. Additionally, previous research has demonstrated that shame influences attitudes toward cosmetic surgery. Specifically, body shame has been associated with greater consideration of aesthetic procedures independent of body dissatisfaction, indicating that body shame has a unique relationship to surgical interest [35, 36]. The psychological postoperative results for individuals undergoing cosmetic surgery appear to be influenced by an individual's preoperative emotional state. In particular, a systematic review concluded that while cosmetic surgery may produce a positive change in self‐esteem or body image in the short‐term, unaddressed shame and emotional pain will likely continue or be shifted to another appearance concern in many individuals undergoing cosmetic surgery and those diagnosed with BDD [37]. These findings highlight the necessity to distinguish between different dimensions of shame when studying emotional processes underlying acceptance of cosmetic surgery. Further research has demonstrated that false self‐presentation increases an individual's level of body shame and their desire to undergo cosmetic surgery [38]. The findings reinforce the difference between internal shame (the evaluation of oneself negatively) and external shame (being perceived by society as being judged negatively) [38]. In addition to the internalized shame mediating the relationship between early adverse experiences and unstable self‐esteem among individuals who pursue cosmetic surgery, it was found that there was a need to understand shame as an emotionally complex construct [39]. Recent studies have also suggested that appearance‐focused social media behaviors and self‐presentation practices may reinforce both internal and external shame processes associated with cosmetic surgery consideration [29, 31]. Furthermore, qualitative studies have also found shame to be a common theme when exploring the experiences of individuals who are pursuing aesthetic surgical procedures; most of the time related to fears of being exposed and seeking validation from society [40].

### 1.3 Context and Rationale of This Study

Lebanon represents a unique sociocultural environment in which cosmetic surgery has become highly visible and socially normalized within everyday life [41]. The post‐conflict development of consumer culture, media exposure, and appearance‐oriented social norms have all promoted the normalization of aesthetic enhancements and the promotion of narrow beauty ideals that promote social comparison and ultimately internalized self‐doubt [41, 42]. Cosmetic surgery is viewed by many as a sign of modernity, social acceptance, and upward mobility among younger adults residing in urban areas [42]. However, the normalization of cosmetic surgery creates emotional tension for individuals who must balance the pressure to appear as aesthetically pleasing as possible while avoiding social judgment or stigma associated with excessive aesthetic modifications [43]. The intersection of these cultural and emotional factors may contribute to the high demand for cosmetic surgery in Lebanon and highlights the necessity for culturally competent psychological assessment. Therefore, the present study aimed to examine whether internal and external shame are statistically associated with the relationship between BDC and acceptance of cosmetic surgery among Lebanese adults. Based on prior literature, it was hypothesized that individuals with higher levels of BDC would report higher levels of shame and greater acceptance of cosmetic surgery, with shame potentially representing one emotional pathway within broader sociocultural and appearance‐related processes.

## 2 Methods

### 2.1 Study Design and Participants

In this cross‐sectional study, data were collected through an online questionnaire distributed via social media platforms and messaging applications. Furthermore, a snowball sampling technique was used to recruit participants to fill out the form. The sample included Lebanese residents who were 18 years and above, of both genders, and regardless of previous cosmetic procedure history. Individuals younger than 18 years and those residing outside Lebanon were excluded. The questionnaire was distributed using Google Forms, and it took around 10–15 min to complete. Participants were given a concise explanation of the purpose of the research, followed by the assurance of anonymity before accessing the questionnaire. Participants were provided with the informed consent electronically, asking them to click on a confirmation statement of participation.

### 2.2 Minimal Sample Size

The G*Power software was used to calculate the minimum sample size. An estimated sample size was 322 participants based on a multiple regression model (*R* ^2^ deviation of zero), an alpha error of 0.05, a statistical power of 0.80, and 9 covariates in the mediation model. A total of 399 respondents served as the final sample, which is above the minimum number and helps increase the statistical power of the analysis.

### 2.3 Questionnaire

The Arabic questionnaire had four parts: (1) sociodemographic data (e.g., age, sex, marital status, income, professional status); (2) the Acceptance of Cosmetic Surgery Scale (ACSS) to evaluate attitudes toward cosmetic surgery; (3) the Dysmorphic Concern Questionnaire (DCQ), a screening measure of subclinical dysmorphic concerns, and (4) the External and Internal Shame Scale (EISS).

#### 2.3.1 Sociodemographic Survey

This section collected sociodemographic information, which included sex, age, educational attainment, marital status, employment situation, monthly income, place of residence, and financial burden. Financial burden [41], body mass index, and the household crowding index were also assessed [44]. This section also examined past aesthetic procedures, current evaluation of treatment options, and future cosmetic surgery intentions among participants.

#### 2.3.2 The Acceptance of Cosmetic Surgery Scale

The Acceptance of Cosmetic Surgery Scale (ACSS), created by Henderson‐King and Henderson‐King in 2005, is used to evaluate people's opinions about cosmetic surgery. The questionnaire has 15 statements, and each one needs to be rated on a Likert scale from 1 (strongly disagree) to 7 (strongly agree), where a higher score represents a higher level of acceptance of cosmetic treatments [45]. There are three subscales in the ACSS: the *Intrapersonal* subscale measures reasons someone may want to fix flaws in their own image, the *Social* subscale assesses motivations related to improving appearance in social or interpersonal settings, and the *Consider* subscale assesses willingness to consider undergoing cosmetic procedures (Cronbach's *α* = 0.91). The ACSS, originally in English, was translated into Arabic using a forward‐backward translation method. First, a professional bilingual translator produced the Arabic version. Then, a second independent translator back‐translated it into English. The research team compared the back‐translated version with the original to identify and resolve any discrepancies. The translation process also received particular attention to ensure conceptual equivalence, given the sociocultural sensitivity of appearance‐related constructs such as shame and cosmetic surgery attitudes (Cronbach's *α* = 0.91).

#### 2.3.3 The Dysmorphic Concern Questionnaire

Validated in Arabic [46], the Dysmorphic Concern Questionnaire (DCQ) is a brief and widely used screening instrument designed to assess preoccupation with perceived appearance‐related defects. The DCQ was originally developed as a screening instrument for dysmorphic concerns related to body image and includes items reflecting cognitive and emotional distress associated with appearance preoccupation. It has 7 items with a 4‐point Likert scale (from 0 (not at all) to 3 (very much)), with higher scores indicating more dysmorphic concern. Internal consistency and predictive validity are high for this scale, especially among clinical and cosmetic surgery groups (Cronbach's *α* = 0.92).

#### 2.3.4 The External and Internal Shame Scale

The External and Internal Shame Scale (EISS) is a scale created to measure trait shame in two domains: in terms of how individuals view themselves (internal shame), and how they believe others view them (external shame). The Arabic version used in this study [47], was previously validated in a Lebanese population and demonstrated good psychometric properties, supporting its cultural applicability within the Lebanese context. It consists of eight items (four in each dimension) that are measured using a 5‐point Likert scale (0 = “Never” to 4 = “Always”) where the greater the value, the higher the degree of shame. Ferreira et al. supported the validity of the EISS and demonstrated its two‐factor composition and high internal consistency [48] (Cronbach's *α* = 0.92 for the total score, 0.87 for external shame and 0.85 for internal shame).

### 2.4 Statistical Analysis

The SPSS v.27 software was used for the statistical analysis. The acceptance of cosmetic surgery score was considered normally distributed since the skewness and kurtosis values were between the −1 and + 1 interval. The Student *t*‐test was used to compare a continuous variable and a dichotomous variable, and Pearson's correlation test was used to correlate two continuous variables. The mediation analysis was performed using PROCESS Macro version 3.4 Model 4, with the number of bootstrap samples set at 5000 and a 95% confidence interval. Four routes result from this analysis: pathway A of the independent variable to the mediator, pathway B of the mediator to the dependent variable, and pathways C and C′ indicating the total and direct effects of the independent variable on the dependent variable. We considered the mediation analysis to be significant if the confidence interval did not pass through zero. Given the cross‐sectional design, mediation analyses were interpreted as statistical associations consistent with indirect effects rather than evidence of causal or temporal relationships. Covariates entered into the model were those that showed a *p* < 0.25 in the bivariate analysis. *p* < 0.05 was considered statistically significant. This threshold was selected in accordance with purposeful variable selection strategies commonly recommended in multivariable modeling to avoid excluding potentially important confounders at early analytic stages [49].

## 3 Results

### 3.1 Participants

In total, 399 participants were included in this study, with a mean age of 25.99 years, and 67.2% were female (Table 1).

**TABLE 1:** Sociodemographic and other characteristics of the sample (*N* = 399).

Variable	*N* (%)
Sex	
Male	131 (32.8%)
Female	268 (67.2%)
Education	
School level	14 (3.5%)
University level	385 (96.5%)
Marital status	
Single, divorced, widowed	354 (88.87%)
Married	45 (11.3%)
Professional status	
Unemployed	212 (53.1%)
Employed	187 (46.9%)
Residence place	
Rural	90 (22.6%)
Urban	309 (77.4%)
Previous aesthetic surgery	
No	288 (72.2%)
Yes	111 (27.8%)

	Mean ± SD
Age (years)	25.99 ± 7.48
Monthly income	1084.35 ± 1546.14
Financial burden	5.25 ± 2.10
Household crowding index	0.95 ± 0.52
Body mass index (BMI)	24.60 ± 4.59
Dysmorphic concerns (dysmorphic concern questionnaire; DCQ)	6.41 ± 5.07
Shame (external and internal shame scale; EISS total)	15.02 ± 6.73
Acceptance of cosmetic surgery	56.66 ± 17.80

### 3.2 Bivariate Analysis of Factors Associated With Acceptance of Cosmetic Surgery Score

Bivariate analyses results are presented in Tables 2 and 3. Females had significantly higher acceptance of cosmetic surgery. Moreover, higher BDC were associated with higher acceptance of cosmetic surgery.

**TABLE 2:** Bivariate analysis of factors associated with acceptance of cosmetic surgery score.

Variable	Mean ± SD	*p*	Effect size
Sex		**0.043**	0.217
Male	54.08 ± 17.84		
Female	57.92 ± 17.68		
Education		0.793	0.072
School level	55.43 ± 19.29		
University level	56.70 ± 17.77		
Marital status		0.254	0.181
Single, divorced, widowed	56.30 ± 17.66		
Married	59.51 ± 18.84		
Professional status		0.256	0.114
Unemployed	55.71 ± 17.19		
Employed	57.74 ± 18.46		
Residence place		0.231	0.144
Rural	54.68 ± 17.39		
Urban	57.24 ± 17.91		

*Note:* Numbers in bold indicate significant *p*‐values.

**TABLE 3:** Pearson correlation matrix of study variables.

	1	2	3	4	5	6	7	8	9
1. Acceptance of cosmetic surgery	1								
2. Age	0.06	1							
3. Monthly income	0.04	0.38***	1						
4. Financial burden	0.08	0.03	−0.12*	1					
5. Household crowding index	0.02	−0.12*	−0.20***	0.11*	1				
6. Body mass index (BMI)	0.08	0.12*	0.13**	0.03	−0.04	1			
7. Body dysmorphic concern	0.18***	−0.06	−0.02	0.03	−0.11*	0.18***	1		
8. Shame total	−0.01	−0.04	0.05	0.01	−0.03	0.22***	0.56***	1	
9. External shame	−0.01	−0.02	0.03	0.01	−0.04	0.19***	0.51***	0.96***	1
10. Internal shame	−0.004	−0.05	0.06	0.01	−0.02	0.23***	0.56***	0.95***	0.82***

* *p* < 0.05.

** *p* < 0.010.

*** *p* < 0.001.

### 3.3 Analysis of Mediation

**FIGURE 1:**
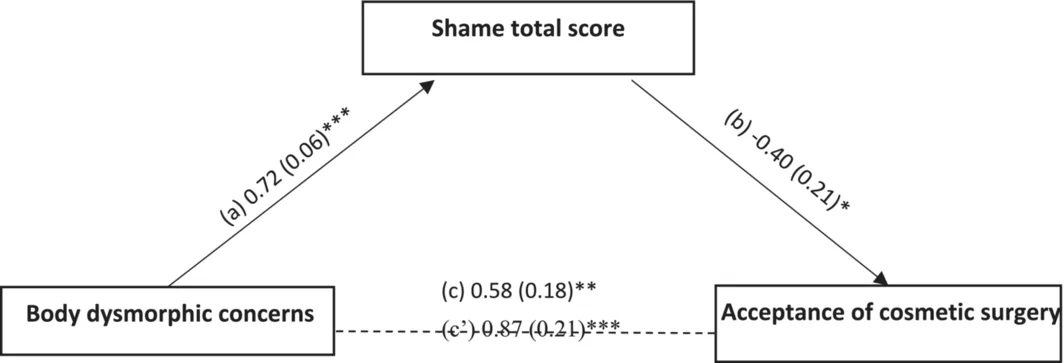
Mediation model taking Shame total score as a mediator. (a) Relation between body dysmorphic concerns (DCQ) and shame (R^2^ = 0.350); (b) Relation between shame and acceptance of cosmetic surgery (*R* ^2^ = 0.083); (c) Total effect of body dysmorphic concerns on acceptance of cosmetic surgery (*R* ^2^ = 0.068); (c′) Direct effect of body dysmorphic concerns on acceptance of cosmetic surgery. The numbers represent regression coefficients and their standard errors. *p < 0.05; **p < 0.01; ***p < 0.001.

**FIGURE 2:**
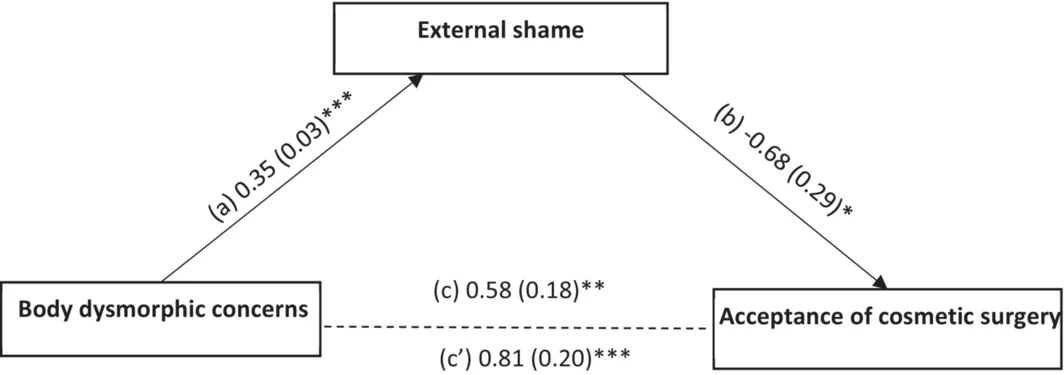
Mediation model taking external shame as a mediator. (a) Relation between body dysmorphic concerns (DCQ) and external shame (*R* ^2^ = 0.284); (b) Relation between external shame and acceptance of cosmetic surgery (*R* ^2^ = 0.081); (c) Total effect of body dysmorphic concerns on acceptance of cosmetic surgery (*R* ^2^ = 0.068); (c′) Direct effect of body dysmorphic concerns on acceptance of cosmetic surgery. The numbers represent regression coefficients and their standard errors. *p < 0.05; **p < 0.01; ***p < 0.001.

**FIGURE 3:**
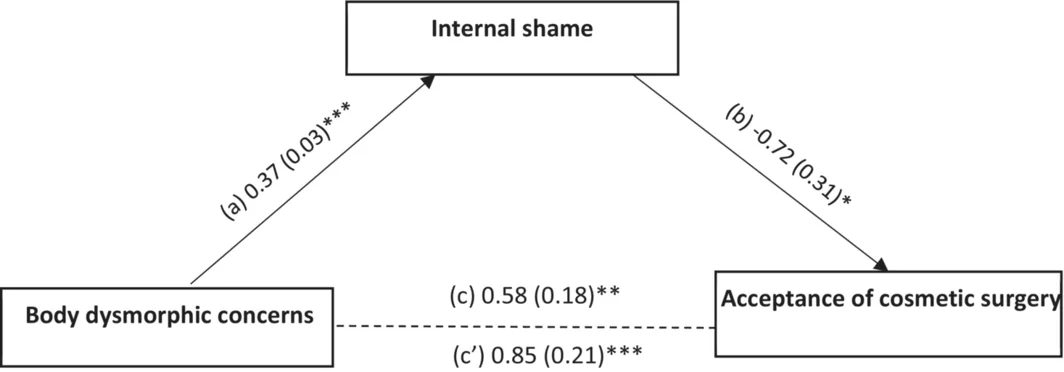
Mediation model taking internal shame as a mediator. (a) Relation between body dysmorphic concerns (DCQ) and internal shame (*R* ^2^ = 0.355); (b) Relation between internal shame and acceptance of cosmetic surgery (*R* ^2^ = 0.081); (c) Total effect of body dysmorphic concerns on acceptance of cosmetic surgery (*R* ^2^ = 0.068); (c′) Direct effect of body dysmorphic concerns on acceptance of cosmetic surgery. The numbers represent regression coefficients and their standard errors. *p < 0.05; **p < 0.01; ***p < 0.001.

The mediation analyses, adjusted for gender, place of residence, age, and financial burden, revealed indirect associations statistically consistent with partial mediation models involving shame. Total shame (indirect effect: Beta = −0.29; Boot SE = 0.13; Boot CI −0.56; −0.05), external shame (indirect effect: Beta = −0.24; Boot SE = 0.11; Boot CI −0.48; −0.03), and internal shame (indirect effect: Beta = −0.27; Boot SE = 0.12; Boot CI −0.52; −0.04) demonstrated significant indirect associations between BDC and acceptance of cosmetic surgery (Figures 1, 2, 3). Higher BDC were associated with higher levels of shame and with greater acceptance of cosmetic surgery, whereas higher levels of shame were associated with lower acceptance of cosmetic surgery (Table 4).

**TABLE 4:** Results of mediation analyses.

Pathway	Beta	Standard error	*p*	95% CI	Effect size
Model 1: total shame score as the mediator					0.50
Total effect	0.58	0.18	0.001	0.23; 0.93	
Direct effect	0.87	0.21	< 0.001	0.46; 1.28	
Indirect effect	−0.29	0.13		−0.56; −0.05	
Model 2: external shame as the mediator					0.41
Total effect	0.58	0.18	0.001	0.23; 0.93	
Direct effect	0.81	0.20	< 0.001	0.42; 1.21	
Indirect effect	−0.24	0.11		−0.48; −0.03	
Model 3: internal shame as the mediator					0.47
Total effect	0.58	0.18	0.001	0.23; 0.93	
Direct effect	0.85	0.21	< 0.001	0.43; 1.26	
Indirect effect	−0.27	0.12		−0.52; −0.04	

## 4 Discussion

The findings of the present study provide insight into the psychological correlates associated with acceptance of cosmetic surgery, particularly the role of shame in the relationship between BDC and cosmetic surgery attitudes. The results were statistically consistent with a partial mediating role of shame in the association between BDC and acceptance of cosmetic surgery. Higher levels of BDC were associated with higher levels of shame, while higher shame was associated with lower acceptance of cosmetic surgery. This pattern suggests the presence of potentially competing psychological processes, in which appearance‐related distress may increase interest in aesthetic change while shame‐related emotional processes may simultaneously contribute to hesitation, avoidance, or fear of judgment regarding cosmetic procedures. These findings are consistent with previous literature describing shame as a psychologically complex and ambivalent emotional factor in appearance‐related decision‐making [40, 50]. Although the explained variance of the mediation models was modest, this is common in psychological and attitudinal research involving multifactorial emotional and sociocultural processes.

### 4.1 Association Between BDC and Shame

Our findings demonstrated that higher levels of BDC were associated with higher levels of both internal and external dimensions of shame. This finding is consistent with Objectification Theory and broader sociocultural models suggesting that repeated appearance‐based comparison and internalization of appearance ideals are associated with increased shame and dysmorphic concerns [25]. Shame appeared to represent an important emotional component associated with the distress experienced by individuals reporting body image dissatisfaction and dysmorphic concerns. The internalization of socially reinforced appearance ideals may contribute to perceptions of bodily inadequacy and self‐evaluative distress [51]. Nevertheless, shame is a self‐derogatory emotion anchored on the fear of being judged poorly and rejected by others; this element is particularly strong when the perceived defect is obvious or stigmatized by society [52]. Santos‐Pereira et al. (2024) found that internal shame was significantly associated with dysmorphic concern and anxiety [53]. Moreover, body image‐related flexibility within psychological practices mediated this relationship, explaining 34% of the variance in dysmorphic symptoms [47]. Similarly, Oliveira et al. (2023) showed that shame not only directly but also indirectly impacted body dysmorphic symptomatology, through mindfulness and cognitive fusion, explaining 56% of the variance in body dysmorphic symptomatology [23, 52]. These findings suggest that shame may interact with broader cognitive and emotional regulatory processes associated with body image disturbances rather than functioning solely as a secondary emotional outcome. Linde et al. (2023) supported this relationship, revealing that Acceptance and Commitment Therapy with a compassion focus (PACT) was associated with reductions in dysmorphic concerns and shame [54], resulting in sustainable enhancements sustained for six months [55]. Overall, these studies support the view that shame is closely associated with dysmorphic concerns and may operate through multiple cognitive, emotional, and sociocultural pathways relevant to appearance‐related decision‐making [52, 55].

### 4.2 Association Between Shame and Acceptance of Cosmetic Surgery

The present findings indicated that higher levels of both internal and external shame were associated with lower acceptance of cosmetic surgery. Internal shame may be associated with reduced acceptance of cosmetic surgery through self‐criticism, hopelessness, or resignation, whereby individuals perceive their perceived flaws as more deeply rooted than surgically modifiable. In contrast, external shame, which reflects concerns regarding negative evaluation by others, may operate through fear of social judgment, exposure, or stigma associated with aesthetic modification. Although both dimensions were associated with lower acceptance, their psychological pathways appear conceptually distinct, suggesting that self‐directed and socially directed shame may influence cosmetic decision‐making in different ways. Studies conducted by Wang et al. (2022) and Taş (2020) similarly indicate that shame may exert a complex regulatory role in cosmetic decision‐making, including avoidance, ambivalence, or relief‐seeking tendencies [56, 57]. These findings may also be interpreted through Self‐Discrepancy Theory and affect regulation models [58]. Shame has been associated with perceived discrepancies between actual appearance and internalized social or idealized appearance standards [54]. Under such circumstances, cosmetic surgery may be perceived as a possible strategy for reducing appearance‐related distress and self‐discrepancy, although this perception may coexist with hesitation, fear of judgment, or avoidance. This pattern is also consistent with an approach–avoidance conflict framework, in which individuals may simultaneously desire appearance‐related change while experiencing emotional processes that inhibit behavioral action [59]. In this study, the inhibitory and ambivalent role of shame highlights the importance of thorough psychological evaluation when an individual consults a cosmetic surgeon. Different dimensions of shame may also be associated with variations in willingness to pursue cosmetic procedures, expectations regarding surgical outcomes, and postoperative psychological adaptation. These findings further support existing recommendations emphasizing the importance of psychological screening and emotionally informed assessment within aesthetic practice.

### 4.3 Association Between BDC and Acceptance of Cosmetic Surgery

Even when the impact of shame was taken into account, BDC remained significantly associated, which suggests that BDC is independently associated with interest in cosmetic surgery. A national survey of Saudi Arabian participants indicated that participants diagnosed with BDD were at a much higher risk of considering cosmetic surgery (*p* < 0.001), regardless of the level of emotional distress [59]. In a study of patients seeking cosmetic surgery in a Chinese plastic surgery clinic, Zhang et al. (2024) found that 11.3% of individuals met diagnostic criteria for BDD, and their interest in surgery was closely associated with dysmorphic symptom severity, regardless of anxiety or depression levels [58]. These findings are consistent with the results of Kaleeny and Janis' (2024) meta‐analysis of 65 cross‐national studies indicating that the prevalence of BDD among surgical patients was 18.6%, with concordant evidence that body dissatisfaction is a primary driver of pursuing surgical procedures despite psychological comorbidity [56]. Collectively, these studies support the notion that BDC is a continuing and autonomous factor influencing the decision‐making process regarding cosmetic surgery. This indicates the need to conduct psychological screening to detect those whose interest in undergoing surgery can be due to distorted self‐perception, compulsive appearance‐driven habits, and emotional distress associated with dysmorphic concerns. Consistent with previous literature, cognitive‐affective disturbances associated with dysmorphic concerns may complicate aesthetic decision‐making and reinforce the relevance of psychological evaluation before cosmetic intervention [51].

### 4.4 Mediation Effect

The findings related to the partially mediating role of shame suggest a psychologically complex and potentially competing process underlying attitudes toward cosmetic surgery. While higher levels of BDC were associated with greater acceptance of cosmetic surgery, higher levels of shame were associated with lower acceptance, resulting in an indirect effect operating in the opposite direction of the direct association. This suppression‐like pattern may reflect an approach–avoidance conflict in which individuals simultaneously experience appearance‐related dissatisfaction and desire for change while also experiencing emotional processes characterized by fear of judgment, exposure, inadequacy, or social rejection. Within the Lebanese sociocultural context, these dynamics may be particularly influential due to the normative and stigmatizing presence of cosmetic surgery. In such settings, individuals may face pressures about appearance, social comparisons, and idealized beauty standards along with concerns about stigma and interpersonal evaluation, thus leading to emotionally ambivalent attitudes toward aesthetic procedures [60]. Furthermore, the relatively modest explained variance observed in the mediation models is consistent with previous research suggesting that attitudes toward cosmetic surgery are shaped by multiple interacting psychological, sociocultural, interpersonal, and economic influences rather than by isolated emotional variables alone.

Recent studies corroborate the influence of shame‐related processes on attitudes toward cosmetic surgery. Wang et al. (2022) found that appearance‐related interactions on social networking sites were positively linked with considering cosmetic surgery via body surveillance and body shame [57]. These findings align with Objectification Theory [25], and support the broader sociocultural framework proposed in the present study, in which appearance comparison, internalization of beauty standards, and perceived interpersonal evaluation may contribute to both dysmorphic concerns and shame‐related emotional processes. In the current findings, both internal and external shame were inversely associated with acceptance of cosmetic surgery, although their psychological mechanisms may differ. Internal shame may contribute to self‐critical beliefs and perceptions of personal defectiveness that reduce confidence in appearance‐related modification, whereas external shame may operate through concerns regarding stigma, social exposure, or negative evaluation by others. Despite these conceptual differences, both forms of shame appeared to converge in limiting overt acceptance of cosmetic surgery within this sample [61].

Similarly, Taş (2020) reported associations between chronic body shame, unresolved trauma, and repetitive cosmetic surgery behaviors among individuals with histories of childhood abuse [62]. These findings further support the possibility that shame‐related emotional processes may complicate cosmetic decision‐making by increasing emotional conflict, dissatisfaction, or persistent appearance‐related distress even in individuals seeking aesthetic change. Such findings also reinforce the importance of psychologically informed and trauma‐sensitive approaches within aesthetic practice [63].

Consistent with these findings, Kazeminia et al. (2023) reported that preoperative emotional factors, including shame and dissatisfaction, were associated with postoperative psychological outcomes following cosmetic surgery [24]. Together, the present findings should be interpreted as reflecting statistically significant but modest associations that may be clinically relevant for psychological screening and risk identification rather than for individual‐level prediction. Overall, the results support the importance of considering both internal and external shame as emotionally relevant dimensions within broader sociocultural and appearance‐related processes associated with cosmetic surgery attitudes.

### 4.5 Clinical Implications

The current findings could have meaningful clinical implications for cosmetic surgery practice and psychological evaluation. The associations we observed indicate that BDC, shame, and attitudes toward cosmetic surgery do not exclusively take place in people who experience these feelings but also in people who undergo surgery. Furthermore, these emotional processes may complicate appearance‐related decision making as well as postoperative expectations. Consistent with existing recommendations within aesthetic medicine, clinicians may benefit from incorporating brief psychological screening tools such as the DCQ and EISS during preoperative consultations to identify individuals experiencing significant appearance‐related distress.

Furthermore, differentiating between internal and external dimensions of shame may help inform more tailored psychological approaches. Internal shame, characterized by harsh self‐evaluation and feelings of personal defectiveness, may be more closely associated with self‐critical cognitions and hopelessness, whereas external shame may be more strongly associated with fears of social judgment and interpersonal evaluation. Accordingly, interventions emphasizing self‐compassion, cognitive restructuring, or emotionally focused psychological support may be beneficial in addressing appearance‐related distress in some individuals considering cosmetic procedures. Within the Lebanese sociocultural context, where cosmetic surgery is both normalized and socially scrutinized, the integration of psychologically informed and culturally sensitive approaches within aesthetic medicine may contribute to more ethically informed and patient‐centered care.

### 4.6 Limitations

Despite the study's contributions, the findings must be viewed with caution due to certain limitations. First, due to its cross‐sectional design, no causation or temporal ordering can be drawn between BDC, shame, and acceptance of cosmetic surgery. Thus, the results should be interpreted as being statistically consistent with mediation rather than evidence of directional or causal paths. It is possible that shame and dysmorphic concerns have a reciprocal or bidirectional relationship. Longitudinal and prospective research is needed to better clarify the temporal dynamics of these psychological processes over time.

Second, the use of self‐report measures may have introduced response bias, including social desirability effects and underreporting of psychological distress. Although validated measures such as the DCQ and EISS were used, constructs such as shame may carry different cultural meanings depending on the sociocultural context, particularly in societies where appearance and social evaluation are highly emphasized. While the Arabic versions of the scales underwent translation and pilot testing procedures, future psychometric validation across broader Lebanese populations would further strengthen confidence in their cultural applicability and construct equivalence.

Third, the sample was predominantly young, urban, and highly educated, which may limit the generalizability of the findings to older adults, rural populations, or individuals with different socioeconomic and educational backgrounds. In addition, snowball sampling through social media platforms may have introduced selection bias and homophily, as individuals with greater interest in appearance‐related topics may have been more likely to participate.

Finally, several variables known to be associated with BDC and cosmetic surgery attitudes, including social media use, appearance comparison, and internalization of beauty ideals, were not directly assessed in the present analyses. The omission of these variables may limit the explanatory scope of the findings and raises the possibility of omitted variable bias. Future research would benefit from incorporating broader sociocultural and digital appearance‐related variables in order to better understand the complex psychological mechanisms underlying cosmetic surgery attitudes.

## 5 Conclusion

The present study contributes to the growing literature examining psychological factors associated with cosmetic surgery attitudes among Lebanese adults. Higher levels of BDC were associated with greater acceptance of cosmetic surgery, while shame was associated with lower acceptance, suggesting the presence of emotionally complex and potentially competing appearance‐related processes. The findings were statistically consistent with a partial mediating role of shame, indicating that individuals experiencing dysmorphic concerns may simultaneously experience appearance‐related dissatisfaction and emotional processes characterized by fear of judgment, inadequacy, or social evaluation. These findings highlight the potential relevance of psychologically informed assessment within aesthetic medicine, particularly in sociocultural environments where cosmetic surgery is both normalized and socially scrutinized. Although the observed associations were modest and should not be interpreted causally, the study contributes to understanding how emotional and sociocultural processes may interact in shaping attitudes toward cosmetic surgery. Further longitudinal and culturally informed research is needed to better clarify these relationships and their implications for clinical practice.

## Author Contributions

F.F.‐R., S.O., G.V.T., and S.H. designed the study; G.V.T. drafted the manuscript; S.H. carried out the analysis and interpreted the results; G.V.T. and G.‐A.A.T. collected the data; K.I. and P.L. corrected the paper for intellectual content; all authors reviewed the final manuscript and gave their consent.

## Funding

The authors received no specific funding for this study.

## Ethics Statement

Ethical clearance was obtained from the ethics committee at the Notre‐Dame des Secours University Hospital (CR:1/2025). When filling out the online form, each participant provided written informed consent, as did their parents or legal guardian(s) if they were study participants under the age of 16. Every step of the process was carried out in compliance with all applicable laws and rules (in accordance with the Declaration of Helsinki).

## Consent

The authors have nothing to report.

## Conflicts of Interest

The authors declare no conflicts of interest.

## Data Availability

The datasets generated and/or analyzed during the current study are not publicly available but are available from the corresponding author on reasonable request.
